# Towards the Establishment of a Porcine Model to Study Human Amebiasis

**DOI:** 10.1371/journal.pone.0028795

**Published:** 2011-12-21

**Authors:** Fabienne Girard-Misguich, Juliette Cognie, Mario Delgado-Ortega, Patricia Berthon, Christelle Rossignol, Thibaut Larcher, Sandrine Melo, Timothée Bruel, Roseline Guibon, Yan Chérel, Pierre Sarradin, Henri Salmon, Nancy Guillén, François Meurens

**Affiliations:** 1 Institut Pasteur, Unité Biologie Cellulaire du Parasitisme, Paris, France; 2 INSERM U786, Paris, France; 3 Université de Versailles Saint-Quentin-en-Yvelines, Département de Biologie, Versailles, France; 4 Institut National de la Recherche Agronomique (INRA), UMR 85, Physiologie de la Reproduction et des Comportements, Nouzilly, France; 5 CNRS, UMR 6175, Physiologie de la Reproduction et des Comportements, Nouzilly, France; 6 Université François Rabelais, Tours, France; 7 IFCE, Nouzilly, France; 8 INRA, UR 1282, Infectiologie Animale et Santé Publique, Nouzilly, France; 9 INRA, UMR 703, Ecole Nationale Vétérinaire Agroalimentaire et de l'Alimentation Nantes-Atlantique (Oniris), Nantes, France; 10 INRA, UE 1277, Plate-forme d'Infectiologie Expérimentale, Nouzilly, France; Royal Tropical Institute, The Netherlands

## Abstract

**Background:**

*Entamoeba histolytica* is an important parasite of the human intestine. Its life cycle is monoxenous with two stages: (i) the trophozoite, growing in the intestine and (ii) the cyst corresponding to the dissemination stage. The trophozoite in the intestine can live as a commensal leading to asymptomatic infection or as a tissue invasive form producing mucosal ulcers and liver abscesses. There is no animal model mimicking the whole disease cycle. Most of the biological information on *E. histolytica* has been obtained from trophozoite adapted to axenic culture. The reproduction of intestinal amebiasis in an animal model is difficult while for liver amebiasis there are well-described rodent models. During this study, we worked on the assessment of pigs as a new potential model to study amebiasis.

**Methodology/Principal Findings:**

We first co-cultured trophozoites of *E. histolytica* with porcine colonic fragments and observed a disruption of the mucosal architecture. Then, we showed that outbred pigs can be used to reproduce some lesions associated with human amebiasis. A detailed analysis was performed using a washed closed-jejunal loops model. In loops inoculated with virulent amebas a severe acute ulcerative jejunitis was observed with large hemorrhagic lesions 14 days post-inoculation associated with the presence of the trophozoites in the depth of the mucosa in two out four animals. Furthermore, typical large sized hepatic abscesses were observed in the liver of one animal 7 days post-injection in the portal vein and the liver parenchyma.

**Conclusions:**

The pig model could help with simultaneously studying intestinal and extraintestinal lesion development.

## Introduction

Amebiasis caused by the parasite *Entamoeba histolytica* can be responsible of severe diarrhoea in humans. Infection with this parasite may be commensally confined to the intestinal lumen without symptoms or can result in invasion of the colonic mucosa leading to ulceration and dysentery. Malnutrition is hypothesized to be one of the host factors influencing susceptibility to infection [1]. Subsequently, the parasites can disseminate via the portal vein to the liver resulting in abscesses [2]. Much of our understanding of the pathogenesis is hampered by the lack of relevant animal models complicated by the fact that trophozoites are destroyed in stomach after oral administration and that the natural stage, the cysts, are not produced *in vitro*.

First investigations to develop an experimental model for intestinal amebiasis have been made on dogs and kittens [3], [4], [5]. Then, rodents replaced dogs and kittens in a search for a suitable animal model. The reproduction of intestinal lesions with *E. histolytica* in an experimental animal model was reported for the first time by Diamond and collaborators [6]. In this study newborn guinea pigs were used. The high level of mortality of newborn guinea pigs infected with *E. histolytica*, was difficult to circumvent. Gerbils were also susceptible to an *E. histolytica* cecal infection but only during the early stages of invasive intestinal amebiasis [7], [8]. Young rats were also used as model of cecal amebiasis [9]. Important lesions were noticed until five days but twenty and thirty days post-infection the mucosa was recovering and amebas were not found anymore. To deepen the analysis of the human intestinal epithelial cell response during *in vitro* interactions with amebas, a SCID mouse-human intestinal xenograft model was successfully developed [10]. This model demonstrated that human intestinal epithelial cells produce inflammatory cytokines in response to an *in vivo* infection. The interaction was studied until 48 hours. However, the nature of the adaptive immune response could not be studied in this model due to the lack of T cells.

In contrast to the well characterized immune response developed during liver abscess, little is known about the protective response in the gut. In 2002, it has been shown that C3H/HeJ mice, with a mutation at the lipopolysaccharide response locus were 60%-infected after intracecal infection, while C57BL/6 or BALB/c mice were resistant [11]. Disease in these mice was limited to the cecum and the morphology of the inflammatory infiltrate was similar to the one observed in humans. This model of resistant *versus* susceptible mice could provide useful clues to the human variability of parasite clearance *versus* invasive disease.

Recently, human colonic explants were used to study host-parasite interactions to determine the kinetics of parasite penetration into the mucus and the mucosa, structural change in the mucosa as well as the development of the inflammatory response [12]. This *ex vivo* model is advantageous to study the first steps of invasion and allows the comparison of different strains with the same colon sample.

In pigs, few species of *Entamoeba* have been identified and among them, *E. polecki* is the best characterized. In wild boars, the prevalence of *E. polecki* and *E. suis* is quite important (17% and 8%, respectively) and these animals are the reservoir of these species [13]. However, it is not clear yet if *E. polecki* is pathogenic for pigs and humans [13], [14], [15], [16], [17], [18]. When man and pig are living in close association with poor sanitation, pig to man transmission of *E. polecki* is considered to be the most likely source of human infection [15]. Pigs are not the natural host for *E. histolytica* but they provide a valuable large animal model for investigating human disease. Indeed, they are closer to human than mouse in terms of genetic, anatomy and physiology [19], [20], [21], [22]. They are similar to humans in size (allowing internal vessels and organs imagery using standard human technologies) feeding patterns, skin structure, renal, cardiac and pulmonary anatomy and physiology [22]. They also have similar gastrointestinal anatomy and function, pancreas morphology and metabolic regulation [22]. Gnotobiotic pigs are available [23] and offer powerful and convenient tools to study the immune response and to manipulate the gut flora. Thus, pigs appear as a potential model for human amebiasis. Few years ago, a study from Variaym and collaborators mentioned the establishment of a noninvasive intestinal amebiasis in gnotobiotic piglets [24].

In a previous study we have shown that polarized porcine cells were susceptible to *E. histolytica* infection with a response clearly oriented toward inflammation and recruitment of neutrophils [25]. Here, we analyzed the ability of pigs to reproduce intestinal amebic lesions. Porcine colonic tissues were first co-cultured with *E. histolytica* to assess interactions between porcine target tissue and the parasite. Then, trophozoites were injected into the large intestine. Additionally, intestinal loops were surgically created in 2 month-old pigs from clean segments of jejunum, and subdivided into consecutive segments designated as loops, allowing the concomitance of infections with several conditions in the same animal during a few weeks. In a similar work in 1985, cecal ulcers were reproduced, in guinea pigs and hamsters using a washed closed-loop of cecum [26]. Unfortunately, animals could not survive more than 72 hours due to ligature of the ileocecal junction. Then, in a further experiment, injection of ameba into the portal vein and liver parenchyma allowed us to initiate the assessment of pigs as potential model to study extra-intestinal amebiasis.

## Methods

### *Entamoeba histolytica* cultures

We cultured *E. histolytica* (virulent HM1:IMSS strain regularly harvested from hamster liver abscesses after 7 days) in complete TY-1-S-33 medium, in 15 ml glass screw cap tubes at 37°C [27].

### Animals and sample collections

All experimental protocols were approved by INRA Committee on Animal Care, and were consistent with the guidelines provided by the French Council for Animal Care.

Four two-month-old female miniature histocompatible SLA^d/d^ pigs and eighteen two-month-old Large White female pigs were used in the study (Table 1). Four histocompatible SLA^d/d^ pigs were sacrificed to collect colon explants. Additionally, six Large White pigs were used to test direct injections of the parasite in the gut; nine Large White pigs for “gut loop” surgeries, and three Large White pigs for direct injections in the portal vein and the liver. Pigs were euthanized by barbiturate overdose after 1–15 days and tissue samples (mesenteric lymph node, jejunal and colon wall, liver) were collected for qPCR and histological analysis. Blood samples were also regularly collected in silicone coated BD vacutainer tubes (BD diagnostic, Franklin Lakes, USA) for serum analysis.

**Table 1:** A total of 22 two-month-old pigs were used in the study.

Direct injections of the parasite in the gut:	*6 Large White female pigs*
“Gut loops” surgeries, parasite injected in the loops:	*9 Large White female pigs*
Direct injections of the parasite in the portal vein and the liver:	*3 Large White female pigs*
Colon explants, parasite co-cultured with colon explants:	*4 histocompatible SLA^d/d^ female pigs*

To collect surgically created gut loops, pigs were euthanized at 1, 4 or 14 days post-surgery and loops were collected. Representative parts of tissues were cut in five 3×3 mm pieces, laid flat, washed with ice cold phosphate-buffered saline (PBS), snap-frozen in liquid nitrogen and stored at −80°C. Then, larger pieces of tissue encompassing “healthy” tissue and lesions were fixed within 4% formalin before paraffin embedding and histological analysis.

Four miniature histocompatible SLA^d/d^ pigs were used for colon explants. These animals have the same histocompatibility complex and immunological parameters did not vary between animals as much as they do in outbred pigs. The porcine colon explant preparation was adapted from a method described by Bansal and collaborators for the collection of human colon [12]. The main difference was the larger size of the collected porcine colon. Twenty large fragments of the proximal colon per pig (10 for culture with parasites and 10 controls) were taken and dissected longitudinally in KREBS medium (117 mM NaCl, 4.7 mMKCl, 1.2 mM MgCl_2_.6H_2_0, 1.2 mM NaH_2_PO4, 25 mMNaHCO_3_, 2.5 mM CaCl_2_.2H_2_O and 11 mM glucose) at room temperature. The tissue was washed 10 times in 200 ml of KREBS. Fragments were cut into segments measuring each 3 cm by 1.5–2 cm and pinned (steel insect pins) with the colon epithelium facing up onto a 4% agarose-KREBS layer (7 ml) in cell culture plates (6 wells). Fresh virulent trophozoites (8×10^5^ in 1 ml of KREBS medium) were added to the luminal face and incubated for 5 minutes at 37°C after which 2 ml of KREBS were added for three hour incubation. Ameba free segments (n = 10) served as controls for each experiment. Three hours after, the supernatants were collected to measure the release of Lactate DeHydrogenase (LDH) (see [25]) and the fragment was plunged for fixation for 2 weeks in a 4% formalin solution before paraffin embedding.

### Experimental inoculation of porcine gut, gut loops, portal vein and liver

**Figure 1:**
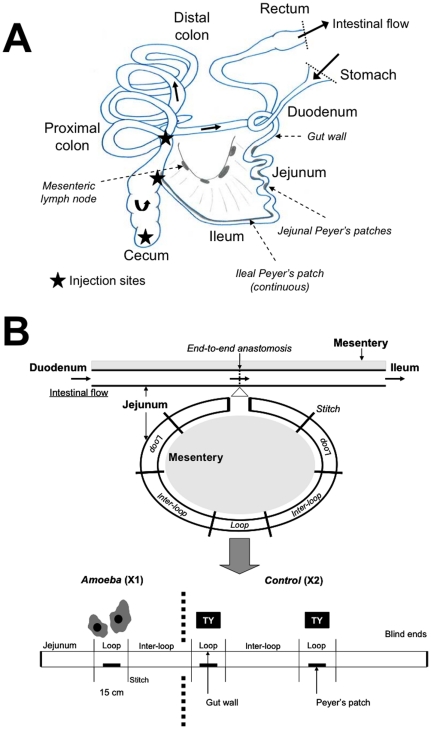
Experimental inoculation of porcine gut and gut loops. **A) Injection sites of** ***Entamoeba histolytica*** **in the pig digestive tract.** A total of 1–3 10^6^ virulent trophozoites in 10 ml TY-1-S-33 medium were injected at three different locations (cecum, ileocecal junction and proximal colon). Four animals were used. **B)** **Schematic representation of jejunal loops**. Three loops were surgically created in a 2–4 m section of the pig jejunum. Proximal loop was inoculated with 1 10^6^ virulent trophozoites in 10 ml TY-1-S-33 medium while distal loops were inoculated only with 10 ml medium. Besides isolated loops intestinal flow from duodenum to jejunum and ileum was maintained in the gut via an end-to-end anastomosis. Blood and lymph flow to the loops were preserved. A total of 9 pigs were used for gut loops.

In four pigs, virulent trophozoites were inoculated directly into the cecum or proximal colon following laparatomy. Of these four animals, one received four 100 ml administrations of an aqueous laxative solution of macrogol 3350 (Colopeg, Bayer, Brétigny-sur-Orge, France) before inoculation of the parasite, and another animal received four daily 100 ml administrations of an aqueous solution of 4% Dextran Sulfate Sodium salt (DSS, Sigma-Aldrich) to induce colitis [28]. Additionally, two pigs were used as negative controls. Briefly, pigs were fasted for 12 hours prior surgery and anaesthesia was induced with intra-muscular (IM) injections of Xylazine (Rompun 5 mg/kg, Bayer, Leverkusen, Germany) and Ketamine (Imalgene 5 mg/kg, Merial, Lyon, France). An endotracheal tube was inserted and anaesthesia was maintained with 1.5–3% isoflurane (Forene, Abott, Queensborough, UK) in 100% oxygen during intermittent positive pressure ventilation with an Ohio V5A mechanical ventilator (Ohio Medical Products, Madison, USA). The pigs were positioned in dorsal recumbence and the ventral abdomen covered with a plastic Steridrape (3 M Health Care, St. Paul, USA). A midline abdominal incision was made and ileocecal junction and cecum were exteriorized. *E. histolytica* (a total of 1–3 10^6^ virulent trophozoites in 10 ml TY-1-S-33 medium) were injected at three different locations (Fig. 1A) using a 10 ml syringe with a 22G needle (Terumo, Guyancourt, France).

To create the loops, a 2–4 m long segment of intestine was surgically prepared in the jejunum, where Peyer's Patches (PP) can be individualized. The surgery was performed in 9 two-month-old pigs (for a complete description of the surgical procedure see [29] and see Fig. 1B for a schematic representation of jejunal loops). This ‘intestinal-segment’ was then subdivided into consecutive segments, designated as ‘loops’ (10–20 cm long, 3 loops), that included a PP, or ‘inter-loops’ without PP (20–100 cm long, 2 inter-loops) (Fig. 1B). Besides isolated loops intestinal flow from duodenum to jejunum and ileum was maintained in the gut via an end-to-end anastomosis. The surgical procedure has been slightly adapted to *Entamoeba* inoculation protocol. Indeed, ingesta was removed from the ‘intestinal-segment’ by flushing once with a warm 100 ml physiological water solution and then again with a warm 100 ml physiological water solution containing Cefotaxime sodium 1 g (Cefotaxime, Sanofi Winthrop, Lyon, France), a third-generation cephalosporin with no action against *E. histolytica*, which was distributed throughout the ‘intestinal-segment’ and left for 30–35 min before an extra warm physiological water solution flush. After flushes, *E. histolytica* (1 10^6^ virulent trophozoites in 10 ml TY-1-S-33 medium) were injected in the proximal loops with 22 G needle (Terumo) while second and third loops were inoculated with TY-medium without parasites (Fig. 1B).

Regarding portal vein and liver injections, three pigs were subjected to a laparotomy as described above for gut injections. Portal vein and liver parenchyma were identified and virulent *E. histolytica* (1 10^6^ virulent trophozoites) were injected in a PBS solution (2 ml) in the portal vein and in PBS and TY solutions in the upper and the lower areas of the liver right lobe using a 10 ml syringe with a 22 G needle (Terumo) in the three pigs. PBS and TY alone were injected in the upper and the lower areas of the left lobe of the two last pigs as negative controls.

Postsurgically, pigs were treated with 20 µg/kg IM three times/day analgesic Buprenorphine (Buprecare, Animalcare, Dunnington York, UK), a semi-synthetic opioid, for 3–14 days, 0.6 to 1 mg/kg three times/day subcutaneous Ketamine (Imalgene, Merial, Lyon, France), and 50 mg/kg IM once/day Cefotaxime sodium (Cefotaxime, Sanofi Winthrop, Lyon, France), for 3–5 days. Pigs were maintained 15 days after *E. histolytica* injection in the gut except for the pig pretreated with macrogol 3350 (Bayer) which was euthanized consecutively to a severe intestinal ileus. Pigs were kept 1–14 days after injections in the loops and 7 days after injection in the portal vein and the liver. Pigs were fed (Sevryplus, Sanders SA, Paris, France) and water *ad libitum* and carefully monitored daily for abdominal discomfort, pain, body temperature, cardiac and respiratory frequency, and the transit of feces.

### Histopathological observation and immunolabelling of amebas in tissue

Formalin-fixed samples (jejunal loops, pieces of colon and liver) were paraffin-embedded. Tissue sections (5 µm thick) were collected onto treated glass slides (SuperFrost Plus, Menzel-Glaser, Braunschweig, Germany) and dried for 2 days at 37°C, then overnight at 56°C, before being deparaffinized and rehydrated. Sections were stained with standard Haematoxylin-Eosin-Safran (HES) to analyze the lesions post-inoculation and with Periodic Acid-Schiff (PAS) to detect the parasite in tissue. For immunolabelling, an antigen retrieval step was performed by autoclaving tissue sections in 10 mM citrate buffer (pH 6.1) for 15 minutes at 121°C. Some trophozoites were immunolabelled with a 1∶100 diluted rabbit anti-serum raised against to internal peptides in the KERP1 protein [30]. For each experiment, a representative image was shown.

### Serum Ig analysis by ELISA

ELISAs were performed on pigs sera and total IgA and IgG concentrations were determined using porcine polyclonal Ig specific kits (Bethyl, Montgomery, USA) according to the manufacturer recommendations. Regarding antibodies specific for *E. histolytica*, a commercial kit for *E. histolytica* serodiagnosis for humans was used (Amibiase H.A.I., Fumouze, Levallois-Perret, France). The assay is based on a hemagglutination reaction, by the specific porcine antibodies present in the peritoneal exudate against sensibilised erythrocytes by the purified Gal/GalNac lectin. The initial dilution of serum or peritoneal exudate was 1∶5 from which twofold serial dilutions were made until a final dilution of 1∶640.

### Quantitative real-time PCR analysis

Quantitative real-time PCR (qPCR) was performed using cDNA synthesized as previously described [23], [25], [31] and following MIQE guidelines [32]. Diluted cDNA (10×) was combined with primer/probe sets and MESA GREEN qPCR MasterMix (Eurogentec, Liège, France) according to the manufacturer's recommendations. The qPCR conditions were 95°C for 30 s, followed by 37 cycles with denaturation at 95°C for 15 s and annealing/elongation for 45 s. To minimize sample variation, we prepared cDNA from same amount of extracted high quality RNA. The quality of RNA was assessed by capillary electrophoresis (Agilent 2100 Bioanalyzer, Agilent Technologies, Massy, France). Real time assays were run on a Bio-Rad Chromo4 (Bio-Rad, Hercules, CA, USA). The expression of two *E. histolytica* transcripts has been assessed using following primers: RPL21 (forward: CCAAACACGTCCAGTCTTTC, reverse: GAGGACATGGACTCTCAAAC; Tm 60°C) and Cysteine Proteinase 5 (CP5) (forward: GGACCATTTGCTGCTATGAC, reverse: CCAGCAACCAACAATCTTCC; Tm 60°C). Primers were designed using Clone Manager 9 (Scientific & Educational Software, Cary, NC, USA) and were purchased from Eurogentec (Liège, Belgium). The correlation coefficients of the standard curves were >0.995 and all qPCRs displayed efficiency between 90% and 110%.

## Results and Discussion

### Co-culture of porcine colon explants with *Entamoeba histolytica*

**Figure 2:**
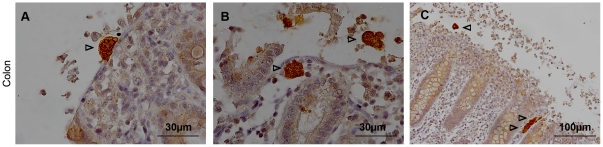
*Entamoeba histolytica* invasion of porcine colon explants. **A)** After two hours of incubation, trophozoites adhere tightly to the colonic mucosa. **B)** At the same time post-inoculation, a disruption of the normal mucosal architecture was observed in areas around the amebas. The amebas are present in the lumen and some are progressing in the tissue. **C)** Four hours post-inoculation, important destruction of the mucosa is observed. Amebas are penetrating in the mucosa.

Before inoculation of the parasites to pigs, we first assessed the interactions of ameba trophozoites with explants of colonic tissue. For that purpose we adapted a protocol previously developed for human colon [12]. Already after 2 hours of incubation, some trophozoites adhered firmly to the colonic tissue (Fig. 2A). In some parts of the epithelial surface, the presence of amebas was observed with disruptions of the normal mucosal architecture. Amebas were observed in the lumen and underlying tissues (Fig. 2B). After 4 hours of incubation, accumulations of material composed of porcine cells detached from the mucosa were observed. The amebas were found within the mucosa (Fig. 2C). LDH enzyme activity was significantly increased in the supernatant from tissues co-cultured with amebas than in controls (P<0.05) (data not shown). After 7 hours of interaction the lysis of the tissue was too important to analyse the results (data not shown). As observed in humans, the parasites were able to rapidly adhere to the mucus layer and the epithelial cells, and to invade the submucosa. Overall, porcine colon explants react similarly to human colon explants [12] with the real advantage to be more available.

### Consequences of single injection of *Entamoeba histolytica* in the porcine intestinal tract

A total of four pigs received luminal injections of *E. histolytica* in the gut (Table 2 and Fig. 1A). After 1–15 days, no macroscopic lesions were observed at the necropsy. However, in one pig out of four, total serum IgA levels were increased after 15 days comparatively to the levels measured at the inoculation time in the same pig and in the controls (data not shown). Furthermore, in another pig, some ameba transcripts were also detected (RPL21 and CP5 mRNA) in intestinal samples after 15 days indicating persistence and/or multiplication of the parasite in the gut (Table 2). This observation is consistent with a previous study showing noninvasive ameba infection in gnotobiotic piglets five weeks post-inoculation [24]. Pigs were maintained two weeks in experimental units maybe not enough to allow tissue invasion and lesion development. It has been reported that ameba can establish infections years after cyst ingestion [33].

**Table 2:** Summary of the main results obtained after *in vivo* experiments.

Injection site(s)	Analyses and results	
**1) In the large intestine: cecum, ileocecal and proximal colon** **(n = 6)**	**A) 1 day post-injection and laxative administration (n = 1):**	
	*-Nothing observed except two necrotic sections probably consecutive to intestinal ileus: 1/1*	
	*-Total seric IgA concentration and ameba qPCR not performed*.	
	**B) 15 days post-injection (n = 3):**	
	*-Macroscopic lesions: 0/3*	
	*-Microscopic lesions: 0/2, no analysis for the third pig*	
	*-Increase in the total seric IgA concentration: 1/3. No detection in the DSS administered pig*.	
	*-Ameba detected by qPCR in cecal sample: 1/3. No detection in the DSS administered pig*.	
	*(2 pigs we also used as controls without injection)*	
**2) In jejunal loops** **(n = 9)**	**Day(s) post-injection:**	
	**1**(n = 2)	*-Macroscopic lesions: 0/2*
		*-Ameba in the lumen: 2/2*
		*-Microscopic lesions: 0/2*
	**4**(n = 1)	*-Macroscopic lesions: 0/1*
		*-Ameba in the lumen: 1/1*
		*-Microscopic lesions: 1/1 (inflammation, oedema and hemorrhages)*
	**7**(n = 1)	*-Macroscopic lesions: 0/1*
		*-Ameba in the lumen:1/1*
		*-Microscopic lesions: 1/1 (inflammation, oedema and hemorrhages)*
	**11**(n = 1)	*-Macroscopic lesions: 0/1*
		*-Ameba in the lumen:1/1*
		*-Microscopic lesions: 1/1 (inflammation, oedema and hemorrhages)*
	**14**(n = 4)	*-Macroscopic lesions: 1/4*
		*-Ameba in the lumen: 2/4*
		*-Microscopic lesions: 2/4*
		*(massive hemorrhage and edema in the mucosa, the sub-mucosa and the serosa in one*
		*pig). Severe acute ulcerative jejunitis. Trophozoites detected in the mucosa, submucosa*
		*and in blood vessels*
		*-Ascitis and serum positive for anti-Gal/GalNac IgG: 1/4*
		*-Ameba detected by qPCR: 2/4*
**3) In portal vein and liver** **(n = 3)**	**-7 days post-injection:** *1/3 pigs presented two massive and small macroscopic abscesses in the liver while 1/2 pigs without macroscopic abscesses presented only a small hepatomegaly*	

*Entamoeba histolytica* trophozoites injection sites were associated to three main approaches, two concerning the intestine and one the liver.

### Acute jejunitis following *Entamoeba histolytica* injection in porcine jejunal loops

After an unsuccessful attempt to establish upon laparotomy obvious intestinal amebic lesions in the porcine cecum or colon we chose to utilize surgically isolated jejunal loops. The main differences between these approaches were the absence of transit in the loops, alteration of the microbiota, and the higher concentration of trophozoites in isolated loops. During the surgery, multiple smooth washes associated to an antibiotic treatment leading to a drastic reduction of the microbiota were performed before trophozoite injection. These features could advantage and/or accelerate tissue invasion by *E. histolytica*. Indeed, it is known that small alterations of the mucosal barrier, the microbiota and the equilibrium between both can predispose to infections. The jejunum was chosen to prepare the loops as it was easier to manage loops in that segment than with the porcine colon. The particular anatomy of this portion of the gut and the difficulties to obtain a tight anastomosis with large intestine epithelium accounts for these issues. Colon and liver are the main targets of the parasite but cases of small bowel [34], and stomach [35] amebomas were also described.

**Figure 3:**
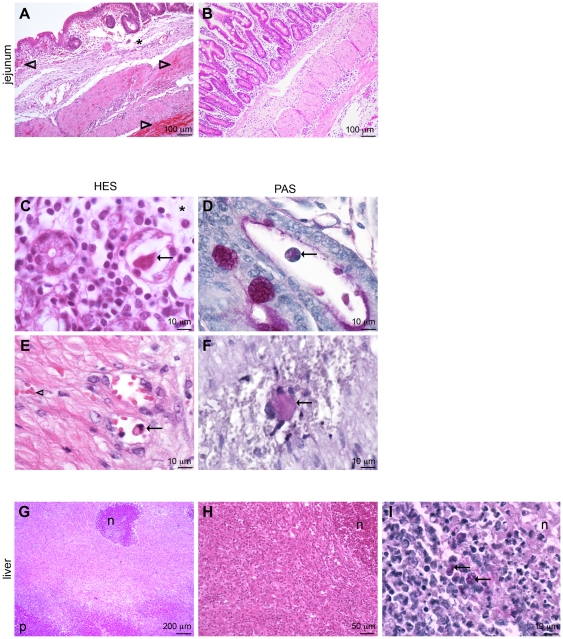
Representative histology of tissues from swine infected with *Entamoeba histolytica* in isolated intestinal loop or after injection in the portal vein and the liver. (A–F except B showing mock jejunum) In jejunum, edema (*) and hemorrhages (arrowheads) were present in mucosa and musculosa. A few amebas (arrows), observable in HES and PAS staining, were observed in crypts of Lieberkühn (C, D), blood vessel (E) and hemorrhagic area (F). (G–I) In liver, some necrotic foci (n) were randomly scattered through the parenchyma (p). Necrotic center of these foci attracted many neutrophils and were surrounded by mononuclear cells and some fibrous tissue at the periphery. (A, B, D, E) 14 days, (C, F) 4 days, and (G–I) 7 days after inoculation.

Nine two-month-old pigs received amebas in their gut loops (Table 2). Loops were collected from one day to two weeks after surgery; there was no evidence at the macroscopic or histological level of altered lymph or blood flows in control loops. The development of the lesions was progressive. Indeed, one day after injection (n = 2 pigs) of the trophozoites, no macroscopic lesions were observed although numerous trophozoites were identified in the lumen (data not shown). While macroscopic lesions were still not observable, histological lesions after HES staining were observed 4 days after injections (n = 1 pig) in loops. Some extravasated red blood cells corresponding to hemorrhages associated to the presence of amebas were detected in the mucosa and the submucosa of the jejunum (Fig. 3C). Trophozoites were also clearly identified in the lumen of some Lieberkühn crypts that were sometimes dilated with necrotic debris (Fig. 3C). These lesions were accompanied by a marked inflammatory response mainly composed of eosinophils scattered all along the mucosa and the submucosa and some neutrophils (Fig. 3A–F).

**Figure 4:**
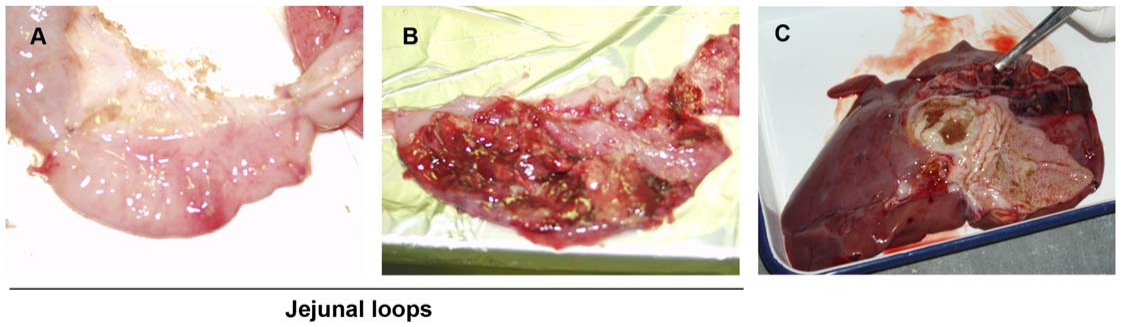
Porcine jejunal and liver macroscopic lesions after injection with *Entamoeba histolytica*. **A)** Fourteen days post-inoculation no lesions were seen in jejunal loops inoculated with TY medium. **B)** On the contrary, in loops inoculated with virulent wild type amebas, a severe acute ulcerative jejunitis was observed associated with large hemorrhagic lesions. **C**. Seven days post-injection, large sized abscess was observed in porcine liver, easily recognizable, with a large cavity filled with yellow-brown fluid.

After 7 and 11 days (n = 2 pigs), obvious macroscopic lesions were still not observed in the two inoculated loops. After 14 days (n = 4 pigs), one out of four inoculated pigs showed severe acute ulcerative jejunitis (Fig. 4B). No lesion was detectable in the control loops inoculated with TY medium (Fig. 4A). Histologically, TY solution inoculated loops appeared mildly edematous without other significant changes. Lesions were strikingly more severe in the infected tissue (Fig. 3A) than in mock jejunum (Fig. 3B). Large hemorrhages and severe edema of the mucosa, the submocusa and the serosa were observed (Fig. 3A). Comparing global villosity morphology of infected or not infected jejunal loops, we identified focally a marked atrophy of the villy at the vicinity of ulcerative lesions. Regarding the parasite, it was possible to identify trophozoites in blood vessels (Fig. 3E) and in the lumen of Lieberkühn crypts (Fig. 3D). Furthermore, trophozoites randomly scattered in the wall of the intestine were observed (data not shown). After 14 days acute peritonitis was observed in the same pig. The detection of peritoneal exudate during the development of intestinal lesions shows a transparietal involvement of the gut by inflammation and the accumulation of inflammatory exudates in the abdominal cavity. We analysed peritoneal exudate from the pig presenting jejunitis and peritonitis for the presence of immunoglobulins directed against *E. histolytica* and significantly higher specific IgG titers were detected in this animal than in controls. Similarly, at the end of the 14 days, in serum, titers of IgG Gal/GalNac anti lectin were significantly higher than at the inoculation time (Table 2). These observations suggest an induction of antibody secreting-cells in the mucosa and of a systemic immune response. In parallel, we noticed for all jejunal loop trials a marked mesenteric lymph node (MLN) enlargement and a marked follicular activation with lymphoid hyperplasia in the presence of the parasite in the loops compared to MLN of control pigs (data not shown). This hyperplasia confirmed the induction of mucosal and systemic immune responses. The hyperplasia and the increases in antibody titers were accompanied by the detection of *E. histolytica* transcripts in the cDNA produced from jejunal tissue (Table 2). In one out of the three other pigs, *E. histolytica* transcripts, trophozoites in the lumen and microscopic lesions (hemorrhages, edema) were detected. The two last pigs (14 days post-injection), negative for qPCR, did not present obvious lesions and were not further analyzed. In the three pigs we observed a thickened gut wall secondary to mild diffuse edema.

The amebic lesions in the porcine intestine were obtained after injection of axenically cultivated *E. histolytica*. However, we found that trophozoites should be freshly isolated from experimental hamster liver abscesses before inoculation in the porcine jejunal loops. Small differences in the trophozoite batches could explain some differences between pigs. Additionally, clinical manifestations of amebiasis in human are highly variable ranging from asymptomatic carriage of the parasite to a possibly lethal, fulminant colitis. In humans, major histocompatibility complex restriction could be a pre-disposing genetic factor that biases the host tissues towards an *E. histolytica*-induced Th2 response [36]. In support of this possibility, the human MHC class II allele DQBl*0601 was associated with resistance to ameba [37]. Similar reasons could explain why we observed different manifestations after inoculations of the outbred Large White pigs with some pigs developing invasive infection and some other pigs not.

### Induction of liver abscesses after injection of *Entamoeba histolytica* in the portal vein and the liver

Three pigs were injected in the portal vein and directly into the right lobe of the liver with virulent wild type amebas (1 10^6^ trophozoites); the first pig presented two large and a few smaller macroscopic foci of necrosis on both diaphragmatic and abdominal surfaces of the right lobes of the liver, 7 days after inoculation (Fig. 4C and Table 2). These foci displayed a central cavity filled with large amount of yellow-brown pus (Fig. 4C). Moreover, adherences were also observed between the gastric wall and the liver (Fig. 4C). These adherences corresponded to peritonitis. Overall the liver was massively increased in size. To exclude a role of PBS or TY in the abscess formation, in the two next pigs, control injections of PBS and TY media in the liver left lobe were performed. These injections failed to induce lesions indicating a role of ameba or ameba products in the development of these necrotico-suppurative foci. In the first pig, histological analyses confirmed the presence of necrotic foci, randomly scattered through the parenchyma (Fig. 3G). Necrotic debris in the centers of these foci attracted many degenerating neutrophils and were surrounded by numerous mononuclear cells and some fibrous tissue at the periphery as observed in figure 3, panels G and H. Furthermore, trophozoites were identified at the periphery of the lesion confirming the involvement of the parasite in the development of the lesion (Fig. 3, panel I). Presence of ameba was confirmed by the detection of RPL21 and CP5 transcripts in a tissue sample (data not shown). Overall, these observations suggest that pigs are sensitive to trophozoites axenically cultivated and could be relevant large animals to study hepatic amebiasis. The first laboratory animal used successfully for hepatic amebiasis was the hamster [38]. Gerbils are also highly susceptible to hepatic amebiasis but progression of liver damage is slower [39] while mice are less susceptible for hepatic amebiasis except mice genetically modified [40].

### Conclusions

With the current study we have shown that outbred pigs can develop both noninvasive and invasive ameba infections with both intestinal and extraintestinal lesions. The pig model could be useful for the study of the pathogenesis of simultaneously early and late intestinal lesions produced by virulent amebas. The ligated intestinal loop model has previously shown its usefulness in studies of intestinal bacterial infections [41], [42], [43], [44]. Here we demonstrate the potential of the model to study the pathogenesis of an eukaryotic pathogen. In the future, this model could be used to characterize lymphocyte subsets recruited following exposure to different strains of *Entamoeba*. As the functional integrity of M-cell antigen uptake in intestinal loops and blood circulation are conserved [29] the study of lymphocyte homing and the description of the subsequent adaptive immune response will be possible. It is known that the outcome of the inoculation varies tremendously depending on the virulence of the strain of *E. histolytica*, host species, nutritional and immune status, intestinal microflora and the presence of concurrent pathogens. With the porcine jejunal loop model, it will be possible to compare various experimental conditions in the same animal, opening attractive perspectives in the understanding of this deadly neglected disease.
